# Metabolomics profiling in hypertension and blood pressure regulation: a review

**DOI:** 10.1186/s40885-020-00157-9

**Published:** 2020-11-15

**Authors:** John O. Onuh, Michel Aliani

**Affiliations:** 1 Center for Molecular and Translational Medicine, Georgia State University, 100 Piedmont Ave SE, Atlanta, GA 30303 USA; 2 Department of Food and Human Nutritional Sciences, University of Manitoba, Winnipeg, MB R3T 2N2 Canada; 3 St. Boniface Hospital Research Centre, 351 Tache Ave, Winnipeg, MB R2H 2A6 Canada

**Keywords:** Hypertension, Metabolomics, Biomarkers, Pathways, Metabolites

## Abstract

Hypertension is a chronic health condition in which blood pressure is usually elevated beyond normal levels. It can progress with serious complications if left undetected and untreated. Incidence of hypertension is on the increase worldwide with debilitating consequences on the health systems of many countries. It is a multifactorial disorder that requires a multi-pronged approach to address it. One such approach is the use of metabolomics or metabolite profiling to understand its underlying cause and possibly control it. Changes in metabolites profiles have been used to accurately predict so many disease conditions in addition to identifying possible biomarkers and pathways associated in their pathogenicity. This will enable their early detection, diagnosis and treatment as well as likely complications that may arise and also assist in development of biomarkers for clinical uses. The objective of this review therefore is to present some of the current knowledge on the application of metabolomics profiling in hypertension and blood pressure control.

## Background

Hypertension is defined as a “condition in which the blood pressure in the arteries is elevated, usually is persistently at or above 140 mmHg of the systolic blood pressure (SBP) or 90 mmHg diastolic blood pressure (DBP) or both” [1, 2]. It has been reported to be a chronic pathological condition that if left unchecked and untreated is a leading risk factor for cardiovascular disease (CVD) and other associated conditions such as stroke, end stage of renal disease and death [3–7]. Approximately 40% of deaths associated with CVD has been reported to be due to hypertensions and the incidence of hypertension is continually growing especially among the low-income population and in developing countries [4, 8, 9]. The incidence of hypertension among adults in developed countries is approximately 24% and it is also assuming an alarming proportion in third world countries [5, 10–12]. Globally, hypertension affects about 1 billion people, with recorded deaths of 9 million annually with China alone accounting for 2.33 million deaths [13, 14].

Hypertension is considered to be a metabolic disorder with the actual causes still unclear though the risk appears to increase with age, usually influenced by unhealthy lifestyles, obesity and physical inactivity [3, 6, 14]. In a majority of cases, it is often associated with dyslipidemia, inflammation and oxidative stress [3]. It is usually regarded as a “silent killer” with no known or visible manifestations of the symptoms and with the majority of hypertensive patients being ignorant of it [15]. Hypertension has been reported to be caused by a complex combination of genetic and environmental factors as well as the interaction between these genetic and environmental factors [16, 17]. Hypertension also develops due to a failure or imbalance in the physiological control of blood pressure (BP) maintained by the nervous system and the kidney [1, 18]. It is associated with the loss of elasticity of the walls of the larger arteries which becomes rigid, creating less space for the flow of blood and leading to increased pressure of fluid [1]. Lifestyles may be a modifying factor in the pathogenicity of hypertension [1, 18]. Consequently, changes in lifestyle patterns are currently being advocated to reduce and control the burden of hypertension and the complications of CVD associated with it [1, 5, 18, 19].

### Current treatment of hypertension

Several pharmacological approaches are presently in use for the management and treatment of hypertension. Common antihypertensive medications include angiotensin converting enzyme (ACE) inhibitors (captopril lisinopril, enalapril), ACE receptor blockers (amlodipine), diuretics (hydrochlorothizide), renin inhibitor (aliskiren) etc. [4]. These antihypertensive drug therapies are effective in decreasing the peripheral vascular resistance and therefore, BP reduction. They target the renin-angiotensin system (RAS) with the aim of effectively lowering BP in patients by controlling the production of angiotensin II or their ability to bind to cellular receptors [1, 20].

### Metabolomics and metabolites profiling

The use of metabolites profiling in hypertension has been generating increasing interest since it was first established that there is an association between serum metabolite profiles and BP in clinically hypertensive patients [20–22]. Several attempts have been made at understanding the association between genetic and metabolic features as well as BP attenuation. This is with a view to discovering biomarkers useful for predicting and diagnosing hypertension using the spontaneously hypertensive rats (SHR) model in comparison with their normotensive Wistar Kyoto (WKY) rats [7, 20, 22–24]. As such, metabolomics study which is mainly focused on low molecular weight compounds that may be present in biological fluids and other tissues may offer useful tool [25–27]. It is a branch of system Biology committed to the investigation of in vivo changes in metabolites profiles associated with drug toxicity, disease processes and gene at different stages [22, 28]. Common examples of these compounds are peptides, amino acids, nucleic acids, carbohydrates, organic acids, vitamins, alkaloids, minerals including other chemical substances that can be produced or broken down in the body [29].

According to [29], the application of metabolomics has previously been focused on clinical and pharmaceutical studies, drug discovery and assessment, clinical toxicology and chemistry. It is a promising tool to helps us to gain new understandings regarding metabolic changes that are associated with the development of disease conditions especially hypertension [3]. Metabolomics profile investigations are being increasingly used to determine small molecules or metabolites that can be considered as potential biomarkers of oxidative stress, inflammation, CVD and a host of other health conditions [3, 29, 30]. Changes in several metabolites have previously been associated with hypertension in several animal models, especially spontaneously hypertensive rats [31, 32]. It is however known that metabolomics studies in humans does not usually correlate with rodent models of hypertension [33]. This may be due to differences in diet, essential nutrients, and metabolic pathways between between humans and the different species of rodents [34, 35].

### Metabolites profiling in hypertension and oxidative stress

Metabolomics approach has also been applied to the study of biological perturbations responsible for hypertension and oxidative stress (Table 1). Most of these studies used the SHR model since its pathophysiological processes closely resemble those of human essential hypertension [20, 22, 23]. The SHR progressively develops hypertension with age, involving several pathological complications such as cardiovascular, cerebrovascular and renal failures when compared to its normotensive (control) WKY rats [22, 23, 26]. The BP may steadily rise with age to 200–250 mmHg, and as such, understanding the metabolic changes responsible for these BP elevations is highly paramount [23]. Although several works have been reported with regards to metabolites profile changes in hypertension, the pioneering work in this area was done by [21]. Using the serum profiles of patients with normal (≤ 130 mmHg), borderline (131–149 mmHg) and high BP (≥ 150 mmHg) obtained by ^1^H NMR, they were able to determine the relationship between metabolic profiles of serum and hypertension. The PCA showed clear differences between serum profiles of patients with low/normal SBP and that of the patients with borderline or high SBP but not between samples of borderline and high SBP. Using this application, they were also able to differentiate the serum samples of normal SBP from borderline and high SBP samples. However, ‘normal’ borderline and the high SBP samples were not different from each other in their serum metabolic profiles. The results also demonstrated that the relationship between serum metabolic profiles and BP were partly due to differences in lipoprotein particle composition between the samples. Also, metabolic changes in serum related to BP were observed in NMR profile even before the SBP reached the defined hypertension values suggesting that the current definition for hypertension may actually be too high [21].

**Table 1:** Summary of studies on the application of metabolomics as a tool for predicting hypertension

S/N	Samples	Sample size	Methodology	Results	References
1.	Spontaneously hypertensive rats (SHR) and normotensive controls, Wistar Kyoto rats (WKY)	6 SHR and WKY each (total *n* = 12)	^1^H-NMR-based urine metabolomics	There was clear separation in the principal components between the 2 samples. This was attributed to comparatively low levels of citrate, α-ketoglutarate, and hippurate, and high levels of creatine, creatinine, and some other metabolites in the urines of SHR than WKY.	[7]
2.	Spontaneously hypertensive rats (SHR) and their aged-matched Wistar Kyoto rats (WKY)	6 SHR and WKY each (total *n* = 12)	^1^H-NMR-based 24 h urine metabolomics	Major metabolic changes were observed for the rats including differences in citrate, a-ketoglutarate, succinate, hippurate, phenylacetylglycine, p-cresol glucuronide, creatine, taurine, and medium chain dicarboxylates	[23]
3.	Spontaneously hypertensive rats (SHR) and their aged-matched Wistar Kyoto rats (WKY)	29 SHR and 18 WKY	LC-QTOF-MS of urine and plasma metabolomics	Partial least squares of several metabolites found in the urine and plasma samples of rats (symmetric dimethylarginine, N2-acetyl-L-ornithine, buthionine sulfoximine, uric acid, α-tocopherol succinate, L-isoleucine, creatinine, and phospholipids) were significantly correlated with hypertension.	[26]
4.	Patients with low/normal systolic blood pressure (SBP ≤130 mmHg), borderline SBP (131–149 mmHg) and high SBP (≤150 mmHg; *n* = 17)	SBP ≤130 mmHg, *n* = 28; SBP (131–149 mmHg, *n* = 19; SBP (≤150 mm; Hg, *n* = 17; Total *n* = 64	^1^H-NMR of serum metabolites	The PCA showed clear differences between serum profiles of patients with low/normal SBP and that of the patients with borderline or high SBP but not between samples of borderline and high SBP. These differences were attributed to differences in serum lipid moieties between the groups.	[21]
5.	Uppsala Longitudinal Study of Adult Men Cohort	504 individuals	GC-MS of plasma	Plasma levels of ceramide, triacylglycerol, total glycerolipids and oleic acid directly correlated with longitudinal changes diastolic BP while cholesterylester levels on the other hand inversely correlated with longitudinal diastolic BP change. However, two glycerolipids validated in an independent cohort. These results suggest a pathophysiological pathophysiological pathways of hypertension	[35]
6.	Chinese Municipal Cohort Study (CMCS)	460	GC-MS of serum	241 metabolites identified, 26 were significantly different between hypertension and control at baseline and 16 out of these were associated with hypertension after adjusting for BMI, smoking and drinking.	[14]
7.	Sympathetic activity and Ambulatory Blood Pressure in Africans (SABPA)	25	GC-MS, LC-MS of urine	38 metabolites differed greatly between the two blood pressure groups with clear PCA separation. Pathway analysis showed changes in ethanol metabolism.	[36]
8.	Hypertensive and normotensive Uygur patients	250	^1^H-NMR of plasma metabolites	OPLS-DA showed clear separation indicating differences between the two groups. Twelve different metabolites were identified suggesting biomarkers of hypertension.	[37]
9.	Case controlled study of 64 essential hypertension and 59 healthy controls	123	NMR of filtered serum	Several metabolites were identified but six of these (alanine, pyruvate, methionine, arginine, adenine, and uracil) were discovered to significantly differentiate EH from HC cohorts. Arginine was up-regulated and alanine, pyruvate, methionine, adenine, and uracil were down-regulated in EH compared to HC.	[18]
10.	European Prospective Investigation Into Cancer and Nutrition (EPIC)–Potsdam study	1116 (135 cases and 981 non cases) of incident hypertension	AbsoluteIDQ p150 Kits (Biocrates Life Scienes AG, Innsbruck, Austria) based on flow injection analysis tandem mass spectrometry technique	127 metabolites were validated to predict hypertension. Six of these were identified as the most predictive biomarkers of incident hypertension. Up-regulation of serine, glycine, and acyl-alkyl-phosphatidylcholines (C42:4 and C44:3) were associated with higher and down-regulation of diacyl-phosphatidylcholines C38:4 and C38:3 with lower predicted 10-year hypertension free survival.	[3]
11.	Black normotensives from The Atherosclerosis Risk in Communities Study	896 black normotensives including 565 women aged 45–64 years	Untargeted GC-MS and LC-MS based quantification of serum samples metabolites.	204 metabolites were measured during a 4–6 weeks period. 38% of the baseline normotensives measured developed hypertension during a 10 years follow-up period. Up-regulation of 4-hydroxyhippurate, a product of gut microbial fermentation was found to significantly increase the risk of hypertension by 17%. PCA yielded sex steroids, α - amino acids, and branch-chain amino acids with only the sex hormone revealed to be significantly associated with hypertension.	[17]
12.	Participants in The Atherosclerosis in Risk Communities Study	9104 ARIC participants without hypertension at baseline		Serum uric acid was positively correlated with incident hypertension. The association is more pronounced with Blacks than Whites.	[38]
13.	San Antonio Family Heart Study	1192 individuals drawn from 42 families	HPLC combined with tandem MS plasma lipidomics of 319 lipid species	Diacylglycerols, especially DG 16:0/22:5 and DG 16:0/22:6 were significantly associated with incident hypertension during follow-up. Four lipid species, including the DG 16:0/22:5 and DG 16:0/22:6 species were also genetically correlated with hypertension.	[39]
14.	Patients with essential hypertension and its Chinese subtypes ^a^(YDYHS and YYDS)	Controls (*n* = 22); YDYHS (*n* = 31); YYDS (*n* = 29); Total *n* = 82	^1^H-NMR and GC-MS of plasma metabolites	The PCA and PLS-DA showed clear separation between the groups with a little overlap between YDYHS and healthy volunteers. Glucose was found to be markedly increased in both YDYHS and YYDS groups, suggesting abnormality in glucose metabolism.	[16]

^a^YDYHS and YYDS - “Yin-deficiency and Yang-hyperactivity syndrome” and “Yin-Yang deficiency syndrome” respectively

To better understand the pathophysiology of hypertension, the associations of circulating plasma metabolites with changes in longitudinal blood pressure (BP) in two study cohorts (Prospective Investigation of the Vasculature in Uppsala Seniors cohort and Uppsala Longitudinal Study of Adult Men cohort) using LC-MS and GC-MS was investigated [35]. The study measured the associations between changes in levels of metabolites and BP and the clinical BP manifestation for a period of 5 years follow-up. later. The mean baseline systolic and diastolic BPs were reported to be 144/76 mmHg while the change over 5 years duration was reported to be 147.7/75.5 mmHg for systolic and diastolic respectively. It was also discovered these changes in baseline BPs were associated with changes in the levels of the metabolites, ceramide, triacylglycerol, total glycerolipids, oleic acid, and cholesterylester. However, there was no relationship between longitudinal changes in systolic BP or clinical BP manifestation. Of these metabolites, diacylglycerol (36:2) and monoacylglycerol (18:0) were found to be associated with changes diastolic BP in the validation cohort suggesting that these metabolites be associated with the pathophysiological pathways of hypertension [35].

The development of hypertension and age-related changes has also been characterized using plasma samples from the SHRs and its normotensive WKY [22]. Several compounds observed to change significantly are free fatty acids (FFA) (hexadecanoic acid, linoleic acid, and stearic acid), amino acids (threonic acid, tyrosine, tryptophan, threonine, phenylalanine, serine, ornithine, methionine, and 3-hydroxyproline), 3-hydroxybutyric acid, citric acid, creatinine, erythrose, myo-inositol, D-methylglucopyranoside, tocopherol, sitosterol, and nonesterified cholesterol. While FFA were found to be significantly increased in SHR when compared to WKY rats, their levels also increased with age from 10 to 18 weeks in the SHRs, suggesting that FFA are potential biomarkers for hypertension.

Metabolic characteristics of SHRs were similarly investigated for all ages, beginning with the prehypertensive as well as the hypertensive stage in comparison to WKY rats using ^1^H NMR urinary metabolites [7, 23]. In the first study to compare the urinary metabolic profiles of SHR with that of their age-matched WKY rats, many metabolites such as citrate, α-ketoglutarate and hippurate were observed to be significantly changing and their levels were lower in SHR urine when compared to WKY urine [7]. The decreased level of citrate was reported to be probably due to mild acidosis which is an indication of the pathogenesis of BP elevation as well as impairment of Krebs cycle [7]. This may also explain the decrease in the excretion of α-ketoglutarate since it is a decarboxylate intermediate of the Krebs cycle. However, hippurate and trimethylamine-N-oxide were reported to be products of the intestinal microflora. Changes in these metabolites indicate that they may be associated with BP regulation in the SHR [7]. However, in the second study to investigate the urinary metabolic profiles of the SHR across different stages of hypertension, urinary excreted metabolites shown to be characteristic of the SHR are citrate, α-ketoglutarate, succinate, hyppurate, phenylacetylglycine, p-cresol glucuronide, creatine, taurine, and medium chain dicarboxylates [23]. Decrease in Krebs cycle intermediates, especially citrate may be due to acidosis in the SHR at the prehypertensive stage and also during early stage of development of hypertension, suggesting an impairment of the renal energy metabolism in the rats. However, metabolic changes in hippurate, p-cresol and phenylacetylglycine give an indication of a change in SHR gut microflora due to genetic and metabolic factors [23].

Recently, a nested case-controlled study involving 460 individuals that were judged to have optimal blood pressure (120/80 mmHg) at baseline was conducted to explore metabolites that are associated with hypertension [14]. A total of 241 metabolites were detected with baseline levels of 26 of them being significantly different for the hypertension group compared with the control. The metabolites considered to be associated with hypertension include some amino acids (threonine and phenylalanine), carbohydrates, carboxylic acids, phenols, and lyxose (a carbohydrate product of gut microbial fermentation). They suggested that low amino acids and the gut microbiome may be possible biomarkers for hypertension. They identified three metabolic pathways including phenylalanine, tyrosine and tryptophan biosynthesis as well to be responsible for the observed changes in hypertension.

A metabolomics study was also conducted in South Africa to investigate the underlying biological mechanisms responsible for increasing cases of hypertension associated with urbanization of the society especially among black male South Africans [36]. Urinary metabolomics profiles of 25 subjects with gas chromatography mass spectrometry (GC-MS) and liquid chromatography mass spectrometry (LC-MS) revealed several metabolites changes that can be considered biomarkers of hypertension, CVD, abdominal adiposity, liver damage, inflammation and oxidative stress. Also, ethanol metabolism through shifted global NADH/NAD^+^ was believed to be the likely mechanistic pathway. The suggested that alcohol abuse may be a contributing factor to the development of hypertension in this population group possibly through alterations in energy metabolism.

A 1H-NMR serum metabolomics of 256 healthy (99) and hypertensive (157) Uygurs was conducted to discriminate metabolites associated with hypertension as well as the related metabolic pathways involved [37]. Orthogonal partial least square-discriminant analysis (OPLS-DA) of the metabolites showed complete separation of the metabolites based on the 2 groups implying that the serum of hypertensive patients is significantly different compared to that of the healthy volunteers with 12 detected metabolites significantly changing. The amino acids (including valine, alanine, pyroacemic acid, inose, p-hydroxyphenylanalnine and methylhistidine) were reportedly lower in hypertensive patients compared to healthy subjects. However, lipid components (VLDL and LDL), lactic acid and acetone were significantly increased in hypertensive patients compared to healthy individuals. These metabolites were therefore considered potential biomarkers of hypertension among this population group.

Primary or essential hypertension is a silent killer because the symptoms in a majority of the victims are either delayed or not detected early enough [1, 15, 18]. This also creates a problem whereby screening for the condition is also ignored. In order to overcome this challenge, a metabolomics probe of the biochemical changes associated with hypertension that would elucidate the mechanisms involved in essential hypertension was conducted [18]. Filtered serum samples of patients with essential hypertension (64) and healthy controls (59) were used to analyze the NMR metabolomics profiles for metabolites that may be significantly changing in the serum and can be considered possible biomarkers of hypertension. Principal component analysis (PCA) and OPLS-DA detected the metabolites to be changing in the serum samples to be alanine, arginine, methionine, pyruvate, adenine, and uracil. These sets of metabolites were also found to excellently correlate with the blood pressure and are associated in 99% of all the cases of hypertension controls. The metabolites are therefore considered potential biomarkers of hypertension for clinical assessment.

Metabolomics has also been used to investigate incident hypertension in a European Prospective Investigation into Cancer and Nutrition (EPIC) Potsdam study [3]. Here, targeted serum metabolites profiles of randomly drawn subjects with cases of hypertension (135) and non-cases of hypertension were followed for an approximately 10 years. A total of 127 metabolites were analyzed and 6 of these were identified to be most possible biomarkers for development of incident hypertension. Among the metabolites detected, increased levels of serine, glycine, and acyl-alkyl-phosphatidylcholines (C42:4 and C44:3) were associated with increased incidence of predicted hypertension free survival. This may possibly be associated with the anti-inflammatory and anti-oxidative properties of these metabolites which confers protection against development of hypertension [3]. On the other hand, diacyl-phosphatidylcholines (C38:4 and C38:3) were associated with reduced predicted 10-year hypertension free survival. This suggests that these metabolites are useful in predicting cases of incident hypertension especially when combined with known risk markers.

It has been reported that “the human metabolome is a measurable manifestation of gene-environment interaction” [17]. As such, it was considered a possibility to explore this metabolome in order to predict the chances of developing hypertension in a sample population of African American with a very high incidence of hypertension. The serum metabolic profile of subjects from the Atherosclerosis Risk in Communities (ARIC) study with normal blood pressure was analyzed over at baseline. Two hundred and four metabolites were detected in the serum samples of the subjects. Of these metabolites, 4-hydroxyhippurate and a sex steroid was discovered to be positively associated with 17% higher risk of hypertension. 4-hydroxyhippurate is a product of gut microbial fermentation of polyphenols and the mechanistic pathway for this regulation may be through oxidative stress suppression. The study provided novel biomarkers for hypertension that had not been previously reported [17]. In a previous study with same ARIC group, serum uric acid was positively correlated with incident hypertension with the association being more pronounced with Blacks than Whites [38].

Diagnosing and treating hypertension has been a serious concern to health scientists and patients due to the complex nature associated with the disease [39]. However, with the advent of new technological tools as metabolomics profiling, plasma lipid profiles can easily be determined to give a better understanding on the association between lipid metabolites and hypertension. An LC-MS plasma lipidomics profile of 1192 Mexican Americans from the San Antonio Family Heart Study was used to determine the association between the lipid profiles and incident hypertension in patients. They detected diacylglycerols (especially, the DG 16:0/22:5 and DG 16:0/22:6 lipid species) to be significantly associated with systolic, diastolic and mean arterial pressures as well as liability of incident hypertension during follow-up period. Four of these lipid metabolites were genetically correlated with the liability of hypertension. They also concluded that changes in diacyglycerol metabolism were discovered to be independent biomarker of hypertension.

### Future prospects

Hypertension is a complex condition that is caused by genetic and environmental factors as well as the interactions between the gene and environment [17]. Metabolomics profiling provides a veritable tool for elucidating the development of hypertension and associated pathways involved [3]. This could possibly serve as a method of screening for hypertension in the population in order to improve clinical treatment and strategies for prevention since metabolite biomarkers appear early before onset of the condition [3, 17]. It is also envisaged to help reduce the possibility of serious complications in individuals that are at risk of hypertension and associated organ damage that may occur if not diagnosed and treated early enough. However, it is important that future intervention studies be conducted to identify potential target metabolites that could be used to reduce the risk associated with it and mechanism for the envisaged hypertension regulation [14]. Part of this future study approach may include a more detailed physiological measurement of hypertension phenotypes in combination with appropriate control mechanisms [40]. More studies also need to be conducted to replicate these findings in order to enable the scientific communities make a definitive statement on this.

## Conclusions

Hypertension is a chronic health condition with debilitating consequences if not detected and treated early enough. It imposes great financial and other burdens on the patients as well as the entire population. Although pharmaceutical drugs are presently being used for the control of hypertension, understanding the mechanisms involved in its pathogenesis early enough before the onset of physiological complications can mean the difference between life and death. Though it is multifactorial in nature, we have attempted to present information on the applications of metabolomics (metabolite) profiling in hypertension and blood pressure regulation. Changes in metabolites profiles demonstrates the possibility of metabolomics as a tool for distinguishing hypertensive patients from normal BP population. This will be very useful for the early detection, diagnosis and treatment of hypertension and other complications that may arise from it. It will also assist in the development of biomarkers of hypertension and CVD complications.

## Data Availability

Not applicable.
